# Field Level RNAi-Mediated Resistance to Cassava Brown Streak Disease across Multiple Cropping Cycles and Diverse East African Agro-Ecological Locations

**DOI:** 10.3389/fpls.2016.02060

**Published:** 2017-01-12

**Authors:** Henry Wagaba, Getu Beyene, Jude Aleu, John Odipio, Geoffrey Okao-Okuja, Raj Deepika Chauhan, Theresia Munga, Hannington Obiero, Mark E. Halsey, Muhammad Ilyas, Peter Raymond, Anton Bua, Nigel J. Taylor, Douglas Miano, Titus Alicai

**Affiliations:** ^1^National Crops Resources Research InstituteKampala, Uganda; ^2^Donald Danforth Plant Science CenterSt. Louis, MO, USA; ^3^Kenya Agricultural and Livestock Research OrganizationNairobi, Kenya; ^4^Institute for International Crop ImprovementKakamega, Kenya; ^5^AG SCI Consulting, LLCCottageville, SC, USA; ^6^Department of Plant Science and Crop Protection, University of NairobiNairobi, Kenya

**Keywords:** Cassava brown streak disease (CBSD), CBSD resistance, RNAi, siRNA expression, CBSV, UCBSV

## Abstract

Cassava brown streak disease (CBSD) presents a serious threat to cassava production in East and Central Africa. Currently, no cultivars with high levels of resistance to CBSD are available to farmers. Transgenic RNAi technology was employed to combat CBSD by fusing coat protein (CP) sequences from *Ugandan cassava brown streak virus* (UCBSV) and *Cassava brown streak virus* (CBSV) to create an inverted repeat construct (p5001) driven by the constitutive *Cassava vein mosaic virus* promoter. Twenty-five plant lines of cultivar TME 204 expressing varying levels of small interfering RNAs (siRNAs) were established in confined field trials (CFTs) in Uganda and Kenya. Within an initial CFT at Namulonge, Uganda, non-transgenic TME 204 plants developed foliar and storage root CBSD incidences at 96–100% by 12 months after planting. In contrast, 16 of the 25 p5001 transgenic lines showed no foliar symptoms and had less than 8% of their storage roots symptomatic for CBSD. A direct positive correlation was seen between levels of resistance to CBSD and expression of transgenic CP-derived siRNAs. A subsequent CFT was established at Namulonge using stem cuttings from the initial trial. All transgenic lines established remained asymptomatic for CBSD, while 98% of the non-transgenic TME 204 stake-derived plants developed storage roots symptomatic for CBSD. Similarly, very high levels of resistance to CBSD were demonstrated by TME 204 p5001 RNAi lines grown within a CFT over a full cropping cycle at Mtwapa, coastal Kenya. Sequence analysis of CBSD causal viruses present at the trial sites showed that the transgenic lines were exposed to both CBSV and UCBSV, and that the sequenced isolates shared >90% CP identity with transgenic CP sequences expressed by the p5001 inverted repeat expression cassette. These results demonstrate very high levels of field resistance to CBSD conferred by the p5001 RNAi construct at diverse agro-ecological locations, and across the vegetative cropping cycle.

## Introduction

Cassava brown streak disease (CBSD) is a major constraint to cassava (*Manihot esculenta*) production in sub-Saharan Africa. The World Health Organization (WHO) has identified CBSD as one of the world's most serious threats to food security (WHO, 2011). A recent survey conducted in villages of Kenya, Tanzania, Uganda, and Malawi estimated losses due to CBSD at about US$750 million annually (Hillocks and Maruthi, 2015). CBSD is caused by two positive-sense single strand RNA (+ssRNA) viruses: *Cassava brown streak virus* (CBSV) and *Ugandan Cassava brown streak virus* (UCBSV), belonging to the genus *Ipomovirus*, family *Potyviridae* (Mbanzibwa et al., 2009; Winter et al., 2010; Ndunguru et al., 2015). Viruses that cause CBSD are transmitted semi-persistently by the whitefly vector *Bemisia tabaci* (Maruthi et al., 2005; Mware et al., 2009) and can occur as single or dual infections (Mbanzibwa et al., 2011).

For many years, CBSD was geographically restricted to the lower coastal regions of East Africa (Hillocks et al., 2001) before being confirmed as present in Uganda in the mid-2000s (Alicai et al., 2007). Since that time, it has spread to the higher altitude areas of East and Central Africa (Legg et al., 2011, 2015; Patil et al., 2015) and now threatens the large cassava-producing countries of West Africa. The rapid spread of CBSD to reach epidemic proportions in East and Central Africa has been linked to a “super-abundance” of whitefly vectors and associated with the evolving environmental and climatic conditions occurring within the region (Jeremiah et al., 2015).

CBSD symptoms vary depending on the cassava variety, the environmental conditions under which the crop is grown and the CBSD-causing species involved, with CBSV the more virulent of the two species (Winter et al., 2010; Mohammed et al., 2012; Alicai et al., 2016; Ogwok et al., 2016). CBSD leaf symptoms are characterized by feathery chlorosis along veins and chlorotic mottling in older leaves. Stem symptoms appear as brown necrotic lesions with die-back from the shoot tip occurring in severe cases. The most economically important impact of CBSD occurs within the storage roots, where symptoms appear as brown, corky, necrotic lesions. This renders affected roots unusable as a source of food and valueless in the marketplace. With cassava central to food and economic security throughout much of East and Central Africa (Fermont et al., 2010) the impact of CBSD has important implications for smallholder cassava farmers, rural communities and growing industries dependent on cassava as raw material. Development and deployment of CBSD-tolerant/resistant germplasm suited to farmers' needs is therefore essential if the impact of CBSD is to be mitigated (Legg et al., 2011).

The efficacy of RNA interference (RNAi) technology to generate resistance against CBSD was first demonstrated in tobacco by transgenic expression of an inverted repeat construct derived from the UCBSV coat protein (CP) sequence (Patil et al., 2011). Experiments in cassava cultivar 60444 showed that transgenic lines harboring the UCBSV-CP RNAi construct were 100% resistant to the homologous UCBSV after grafting inoculation under greenhouse conditions, with no virus detectable by RT-PCR in leaf and storage root tissues (Yadav et al., 2011). Under confined field conditions at Namulonge, Uganda, the same transgenic lines were found to be 100% resistant to UCBSV (Ogwok et al., 2012), but susceptible to infection by the non-homologous CBSV. The exception was transgenic line 718-01 that expressed the highest level of siRNAs derived from the UCBSV-CP sequence (Ogwok et al., 2012). At harvest, 90% of storage roots from plants of the non-transgenic controls were found to have severe root necrosis as a result of CBSD infection. In contrast, 95% of roots from transgenic line 718-01 were free of necrotic symptoms and neither UCBSV or CBSV was detectable in 95% of the storage roots (Ogwok et al., 2012). In a similar approach, Vanderschuren et al. (2012) reported high levels of resistance to CBSD under controlled growth conditions by expressing a CBSV-CP inverted repeat construct in cassava cultivars 60444 and TME 7.

The Virus Resistant Cassava for Africa (VIRCA) project aims to utilize RNAi to generate and deliver cassava with enhanced resistance to CBSD to farmers in East Africa (Taylor et al., 2012). Proof of concept in cassava cv. 60444 utilized the CP of UCBSV, as this was the only sequence information available for a CBSD causal pathogen in 2008. Access to additional genomic sequence data for CBSV and UCBSV enabled development of an improved RNAi construct in which near full-length CP sequences from both CBSV and UCBSV were fused in tandem and expressed as an inverted repeat driven by the *Cassava vein mosaic virus* (CsVMV) promoter (p5001). Construct p5001 was transformed into the Ugandan, farmer-preferred cv. TME 204 (Chauhan et al., 2015), and high levels of resistance to both CBSV and UCBSV were demonstrated by graft-inoculation experiments in the greenhouse. Data indicated that levels of resistance were positively correlated with levels of transgenically expressed CP-derived siRNAs (Beyene et al., 2016a). Here we report performance of p5001 TME 204 transgenic lines established in confined field trials (CFT) at Namulonge, Uganda and Mtwapa, coastal Kenya under conditions of naturally vector-transmitted CBSD, and durability of resistance to CBSD across the vegetative cropping cycle.

## Materials and methods

### Plant preparation, shipment, and hardening

Production of transgenic plants of cassava cv. TME 204 with the inverted repeat construct p5001 was described previously by Chauhan et al. (2015). Twenty-five p5001 transgenic plant lines carrying 1–2 copies of the T-DNA were prepared and confirmed for accumulating CP-derived siRNAs by Northern blot (Beyene et al., 2016a). The 25 transgenic plants were micropropagated along with non-transgenic wild-type TME 204 plantlets. A total of 100 plantlets per line (event) were established in 50 mL Falcon tubes in a manner reported previously (Ogwok et al., 2012). Plantlets were dispatched from the Donald Danforth Plant Science Center (DDPSC), St. Louis, MO, USA with the appropriate import and phytosanitary documents to the National Crops Resources Research Institute (NaCRRI), Namulonge, Uganda.

Upon receipt at NaCRRI, plantlets were hardened in a biosafety level II screenhouse in 100% humidity at 24–28°C under transparent polythene bags (Ogwok et al., 2012). After 4 weeks of growth in vermiculite, plants were transferred to a mixture of vermiculite and sterilized soil at a 1:2 ratio and maintained for an additional 4 weeks before transfer to the field.

### Establishment of confined field trial at Namulonge, Uganda

A confined field trial (CFT) was established with 30 entries at NaCRRI, Namulonge. The trial consisted of 25 independent transgenic p5001 lines of cv. TME 204, two tissue culture-derived non-transgenic TME 204 entries (WT1 and WT2) produced at DDPSC, one stake cutting-derived entry of TME 204 (WT3), plus two farmer-preferred cultivars NASE 3 (TMS 30572) and NASE 14 entries obtained locally from farmers' fields. To ensure that the materials collected from farmers' fields were free of CMD and CBSD, PCR and RT-PCR were performed prior to planting on leaf samples as described previously (Ogwok et al., 2012).

After 8 weeks of growth in the screenhouse, hardened plants were transferred to the CFT enclosure with an area of 67 × 74 m. Preparation of the field, transfer of plants and planting was performed as described previously by Ogwok et al. (2012). Plants were established in a randomized complete block design (RCBD), consisting of eight replications and a plot configuration of 10 plants per entry (paired parallel rows of five plants each) at a spacing of 1 × 1 m. A single row of cassava stakes of cv. TME 14 infected with CBSD was planted around each plot to ensure maximum disease transmission to the test materials. Prior to planting, the “spreader” TME 14 plants were tested by RT-PCR (Ogwok et al., 2015) to ensure that they harbored both CBSV and UCBSV. After planting in the field, plants were watered daily for 1 week and then three times per week thereafter. No fertilizers or agrochemicals were applied to the plants. Hand weeding was performed as and when required.

### Establishment of confined field trial at Mtwapa, Kenya

A CFT was established at Mtwapa, coastal Kenya consisting of a total of 21 entries. This included 19 transgenic p5001 TME 204 lines and two micropropagated wild-type TME 204 controls. The 19 transgenic p5001 lines were a sub-set chosen from the 25 p5001 lines established in the CFT at Namulonge, Uganda and included both high and low expressers of transgenic CP-derived siRNAs. Establishment of plantlets in 50 mL Falcon tubes at DDPSC, transport, transplanting and hardening of the plants in a biosafety level II screenhouse at Kenya Agricultural and Livestock Research Organization (KALRO) Biotech, Nairobi, Kenya followed procedures as described above (Ogwok et al., 2012). The Mtwapa CFT was established as a RCBD with four replicates of 10 plants each and with a single row of the local cassava cultivar Ex-mariakani showing CBSD symptoms used as spreader row plants.

### Establishment of stake-derived field trial at Namulonge

Plants within the CFT at Namulonge were tested for presence of CBSV and UCBSV at 6.5 months after planting as described by Ogwok et al. (2012). Woody stem cuttings (length ~15 cm) were obtained from CBSD-free plants from three of the eight replicates and used to establish a second trial. This trial consisted of 15 entries, of which 11 were transgenic p5001 TME 204 lines, two non-transgenic local varieties NASE 3 and NASE 14 and two non-transgenic TME 204 (one obtained from wild-type plants of the previous trial and the second from UCBSV/CBSV-free plants collected from a farmer's field). The trial was established in RCBD with four replications, each plot having 25 plants per entry (5 × 5 rows) at a spacing of 1 × 1 m. A single row of cassava stakes of cv. TME 14 infected with CBSD was planted around each plot to ensure maximum disease pressure on the test materials. The same agronomic practices were performed as described above.

### Visual assessment of CBSD symptoms

Plants were visually assessed for CBSD and CMD symptoms beginning 1 month after planting (MAP) and every month thereafter. Severity of CBSD symptoms on shoots and stems of plants within each plot were assessed and scored on a 1–5 scale (Ogwok et al., 2012). Incidence was calculated as the percentage of plants showing CBSD symptoms on leaves and stems per line. Adult whiteflies (*B. tabaci*) present on the underside of the five uppermost, fully expanded leaves of all experimental plants were counted and recorded at Namulonge, Uganda.

In order to assess storage root damage due to CBSD, storage roots were dug up using a hoe and assessed for CBSD necrosis. Plants from three of the eight replicates (30 plants per line) were harvested and evaluated at Namulonge at 6.5 MAP and the remaining five replicates (50 plants per line) were harvested 12 MAP. Plants from the stake-derived CFT at Namulonge were harvested for assessment of CBSD in the root at 8 MAP. At Mtwapa, all plants were harvested and evaluated at 12 MAP. Storage roots were weighed on a digital scale and each root was sliced transversely along their length into five pieces. Each slice was visually assessed for presence and severity of CBSD necrosis on a 1–5 scale (Ogwok et al., 2012; Odipio et al., 2014), adapted from that reported by Hillocks et al. (1996). Storage root CBSD incidence was expressed as (a) percentage of plants per line that showed CBSD symptoms in their storage roots and (b) number of roots per line showing CBSD symptoms expressed as a percentage of total number of roots harvested. Mean severity of CBSD was calculated from the averages of individual root severity scores of the symptomatic roots (scores of 2–5) per plot. The percentage of marketable/usable roots was calculated as the number of roots that showed severity of 1–2 (scale of 1–5) adopting breeders' practices for line selection (Kawuki et al., 2016).

### Sampling of leaf and storage root tissues

Leaf samples were picked randomly from five out of 10 plants in each plot. The central leaf lobe was removed and placed into a paper envelope and stored in a cool box containing ice. Samples were taken to shaded area, removed from the envelope and fastened onto newsprint using masking tape. Sheets of the labeled samples were pressed in a plant press, transported to the laboratory and stored at room temperature until required. For storage root samples, three asymptomatic and three symptomatic roots were picked at random from each plot. A transverse slice was cut from storage roots using a kitchen knife. Samples were wrapped in aluminum foil, labeled and placed in a cool box with ice, transported and stored at −80°C.

### Total RNA extraction from leaf and storage root samples

Five dried leaf samples collected from each plot were pooled to generate one sample of approximately 0.20 g and used for RNA extraction by the CTAB method (Lodhi et al., 1994). For the root samples, ~0.2 g of each was weighed and RNA extracted using the CTAB method. All RNA extracts were treated with DNase I and subjected to cDNA synthesis using the Invitrogen Super-Script® III First-Strand synthesis kit. RT-PCR was performed on the synthesized cDNAs for simultaneous detection of UCBSV and CBSV. Primers CBSVDF2 (5′-GCTMGAAATGCYGGRTAYACAA-3′) and CBSVDR (5′- GGATATGGAGAAAGRKCTCC-3′) (Mbanzibwa et al., 2011) were used to amplify about 437 and 343 bp of UCBSV and CBSV, respectively. PCR products were resolved by electrophoresis on a 1.2% agarose gel and analyzed using a SYNGENE UGENIUS 3 gel documentation system. The results were reported as presence or absence of the virus(es) based on the detectability of the expected PCR signal.

### Analysis of CBSV and UCBSV sequence diversity

In order to perform a virus diversity study, leaf samples were collected from CBSD symptomatic wild-type TME 204 and local cultivars within the CFT site at Namulonge and from within the CFT and nearby field plots at Mtwapa. Leaves were pressed and dried on newsprint as described above, and shipped to DDPSC, St. Louis, MO, USA. Between 0.02 g and 0.05 g dried leaf sample was removed from the pressed newsprint, placed into a 2 ml screwcap tube and ground with a sterile ceramic bead to a fine powder using a FastPrep-24 (MP Biomedical, Solon, OH) set at 4.0 m/s twice for 30 s, followed by addition of 700 μL of cetyltrimethylammonium bromide (CTAB) RNA extraction buffer (Doyle and Doyle, 1990). Genomic DNA was removed using the TURBO DNA-free Kit (Ambion, Houston, Texas) and the resulting RNA run on a denaturing agarose gel to check for quality. RNA was used for cDNA synthesis and amplification of 3′-viral genomic regions of about 1607 bp (CBSV) and 1670 bp (UCBSV) essentially as described by (Ogwok et al., 2015) using degenerate primer combinations CBSVF2 (5′-GGRCCATACATYAARTGGTT-3′) (Mohammed et al., 2012) and CBSVDR (5′- GGATATGGAGAAAGRKCTCC-3′) (Mbanzibwa et al., 2011). PCR products were cloned as described earlier (Ogwok et al., 2015) and sequenced using the Sanger sequencing method. Raw sequence reads were processed using Vector NTI Advance v11.5.3 (Invitrogen, Carlsbad CA, USA). Cleaned sequences were subjected to homology search using BLASTN (Zhang et al., 2000) and BLASTP (Altschul et al., 1997) available at the National Center for Biotechnology Information (NCBI) for virus species identification. Phylogenetic analyses from multiple sequence alignments were produced using MUSCLE (Edgar, 2004) with default settings and phylogenetic trees were viewed and edited using TreeGraph2 (Stover and Muller, 2010).

### Statistical analysis

For CFT established with 30 entries/lines at Namulonge, Uganda, eight replicated blocks were employed in a randomized complete block design. Each entry was represented in plots consisting of two parallel rows, each row with 5 plants, for a total of 10 plants. Data for early season assessment to estimate foliar CBSD incidence and severity scores were collected from all eight replicated blocks. The 6.5 MAP assessment for storage root CBSD incidence and severity was based on data collected from three replicated blocks, each plot having ten plants per line. The 12 MAP data for both foliar and root CBSD incidence and severity of the 30 entries were collected from the remaining five replicated block, each plot consisting of ten plants per entry. Within each plot at harvest (6.5 and 12 MAP) every plant was harvested separately with all roots being segmented and scored for CBSD symptoms on a plant by plant basis. For the CFT conducted at Mtwapa, Kenya with 21 entries, data of foliar and storage root CBSD incidence and symptom severity scores were collected from four replicated blocks with each entry represented in plots by paired rows of five plants each. For the stake-derived CFT planting of 15 entries at Namulonge, Uganda, data for foliar and root CBSD incidence and severity were collected from four replicated blocks, each plot consisting of 25 plants (5 rows, 5 plants per row) per entry. Data for CBSD incidences and severity scores were subjected to Analysis of Variance (ANOVA) using ARM (Gylling Data Management, Inc., Brookings, SD, USA, formerly Agriculture Research Manager Software). Wherever the ANOVA process detected significant treatment (i.e., entry) effects (Calculated *F*-test where *p* ≤ 0.05), separation of means was done using Duncan's new multiple range test (alpha ≤ 0.05).

## Results

### Selection and establishment of transgenic p5001 lines in CFTs

Production of TME 204 plants transgenic for the p5001 RNAi construct, and confirmation of integrated T-DNA copy number was reported by Chauhan et al. (2015). Lines with 1–2 copies of p5001 T-DNA were characterized to determine levels of CP-derived siRNA expression by Northern blot (Beyene et al., 2016a). Transgenic lines expressing low, medium and high levels of siRNAs derived from p5001 were evaluated by challenge with CBSV and UCBSV using a bud grafting procedure for virus inoculation under greenhouse conditions (Wagaba et al., 2013). The level of resistance to these viruses was found to be positively correlated with the levels of CP-derived siRNAs (Beyene et al., 2016a).

To evaluate efficacy of the p5001 construct under whitefly transmitted CBSD under field conditions, 25 independent transgenic lines were planted at Namulonge, Uganda. Within the 25 selected transgenic lines, seven lines expressed low, and the remaining lines expressed medium to high levels, of transgene-derived siRNAs (Table 1, Supplementary Figure S1). *In vitro* plantlets from the 25 transgenic lines, including three non-transgenic entries of TME 204, were used to establish a CFT consisting of eight replications. Each plot contained 10 plants per entry, resulting in 80 plants per line for a total of 2000 transgenic plants. Locally sourced CBSD-free stake cuttings of CBSD tolerant cultivars NASE 3 and NASE 14 were included in the field trial for comparison.

**Table 1:** **siRNA expression levels of transgenic p5001 TME 204 lines tested in confined field trials at Namulonge, Uganda, Mtwapa, coastal Kenya, and in a stake-derived trial at Namulonge, Uganda**.

**Line/Entry**	**Relative expression level (% of control)^a^**	**siRNA expression^b^**	**Tested in Namulonge CFT**	**Tested in Mtwapa CFT**	**Tested in Stake-derived durability Trial**
5001-01	66.9	+++	Yes	Yes	Yes
5001-08	97.3	+++	Yes	Yes	Yes
5001-09	100.3	+++	Yes	No	No
5001-10	99.6	+++	Yes	Yes	Yes
5001-16	7.8	+	Yes	Yes	No
5001-18	82.0	+++	Yes	Yes	Yes
5001-20	28.1	+	Yes	Yes	No
5001-22	56.0	++	Yes	No	No
5001-25	73.7	+++	Yes	No	No
5001-26	72.7	+++	Yes	Yes	Yes
5001-27	34.1	+	Yes	No	No
5001-30	79.7	+++	Yes	Yes	Yes
5001-34	99.3	+++	Yes	Yes	Yes
5001-35	102.5	+++	Yes	Yes	Yes
5001-40	19.9	+	Yes	Yes	No
5001-42	98.6	+++	Yes	Yes	No
5001-45	23.0	+	Yes	No	No
5001-46	78.7	+++	Yes	Yes	Yes
5001-47	79.0	+++	Yes	Yes	Yes
5001-48	10.6	+	Yes	No	No
5001-50	75.3	+++	Yes	Yes	Yes
5001-53	40.1	++	Yes	Yes	No
5001-69	76.7	+++	Yes	Yes	No
5001-76	45.5	++	Yes	Yes	No
5001-77	75.2	+++	Yes	Yes	No
718-01	100.0	+++	No	No	No

a *siRNA expression expressed relative to signal intensity of control line 718-01 (Ogwok et al., 2012) set at 100%*.

b *Ranking of transgenic lines based on siRNA expression levels (+ = low, 0–33.3% of control line 718-01 ++ = medium 33.4–66.6% of control line 718-01 and +++ high, 66.7–100% or more of control line 718-01)*.

### Foliar and storage root CBSD incidence and severity

**Figure 1:**
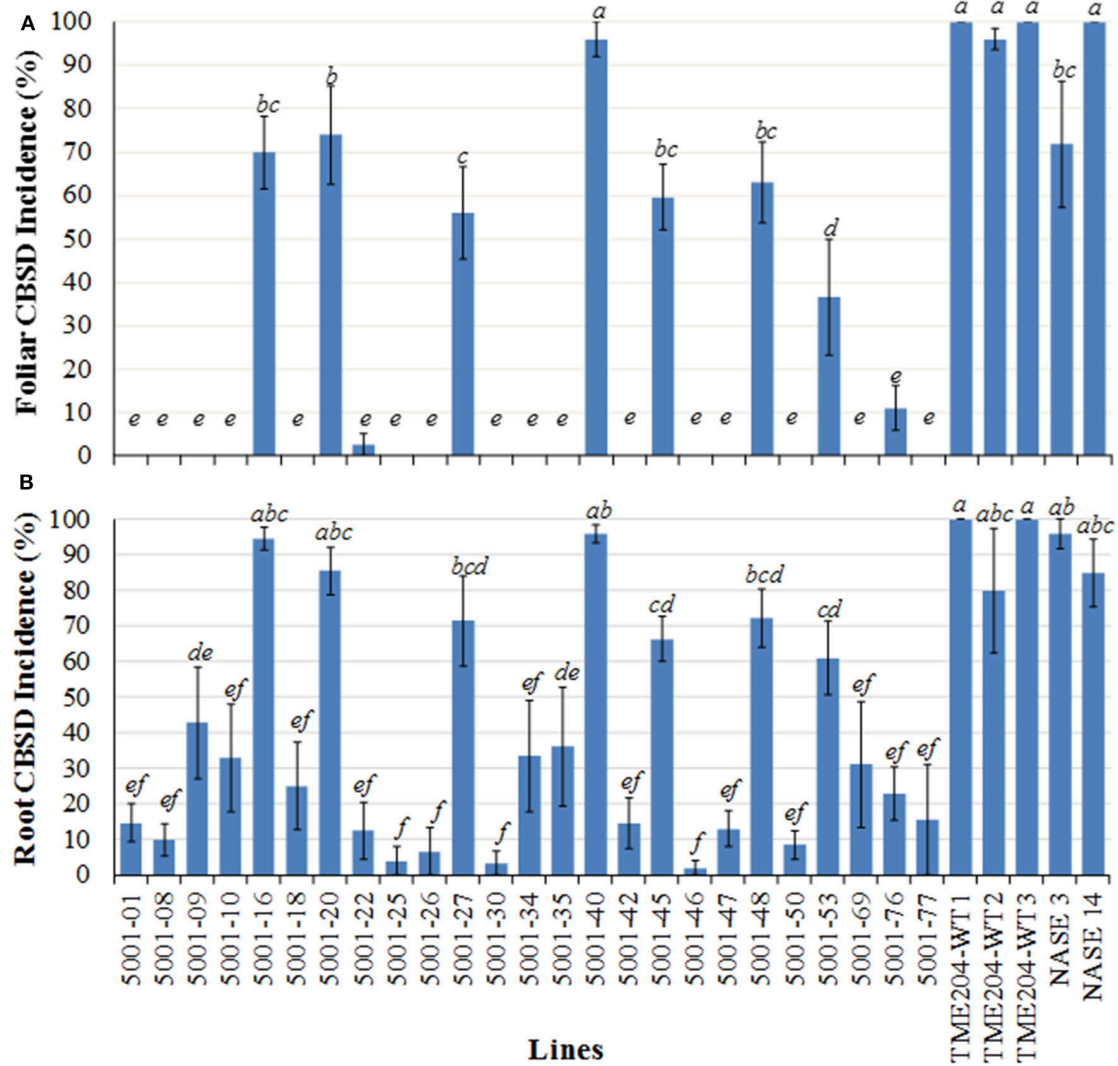
**Incidence of CBSD symptoms on shoots and storage roots of transgenic TME 204 plant lines, non-transgenic TME 204 controls and local cultivars NASE 3 (TMS 30572) and NASE 14 at 12 months after planting within a confined field trial at Namulonge, Uganda**. TME 204 lines were established from tissue culture-derived plants transgenic for inverted repeat construct p5001 that consists of coat protein (CP) genes of *Ugandan Cassava brown streak virus* (UCBSV) and *Cassava brown streak virus* (CBSV) fused in tandem. **(A)** Shoot CBSD incidence and **(B)** storage root CBSD incidence. TME 204-WT1 and -WT2 represent entries of wild-type TME 204 derived from *in vitro* micro-propagated plants. Entries TME 204-WT3, NASE 3, and NASE 14 wild-type plants were derived from disease-free stake cuttings obtained from farmers' fields. Storage roots were sliced transversely five times along their length and visually assessed for presence of CBSD symptoms. Data were collected from replicated plots of 10 plants each and are presented as the average percentage of plants showing CBSD symptoms across the five plots. Approximately five storage roots were assessed per plant. Presence of CBSD within one or more storage roots resulted in a positive score for that plant. Means followed by the same letter are not significantly different (*P* ≤ 0.05, Duncan's multiple range test).

All experimental plants in the field were assessed on a monthly basis for presence and severity of CBSD and CMD symptoms on leaves and stems. CMD incidences on these lines was reported recently (Beyene et al., 2016b). In an initial CFT established at Namulonge, foliar symptoms of CBSD on wild-type TME 204 plants became visible at 2 MAP. All three wild-type TME 204 entries displayed foliar CBSD incidences that reached 96–100% by 12 MAP (Figure 1A, Supplementary Figure S3). Average CBSD symptom severity at 6 MAP ranged from 2.6 to 2.9 (scale of 1–5), increasing to 3.2–3.4 by 12 MAP (Table 1). CBSD foliar incidences were seen to be present on the local control cultivars NASE 3 and NASE 14 at 48 and 90%, respectively, by 6 MAP (Supplementary Figure S3), increasing to 71 and 100%, respectively, by 12 MAP (Figure 1A) and showing average CBSD severity scores of 4.4 and 4.3 at 12 MAP (Table 2). Performance of the transgenic lines contrasted significantly with the wild-type TME 204 and local controls. Sixteen of the 25 TME 204 transgenic lines remained free of CBSD foliar symptoms across the full 12-month growing cycle (Figure 1A, Supplementary Figure S3). The nine transgenic lines that developed foliar CBSD symptoms did so at incidences that ranged between 2.5% (5001-22) and 96% (5001-40). No new transgenic lines developed foliar CBSD symptoms between 6 and 12 MAP. However, as seen for the non-transgenic TME 204 controls (Figure 1A; Supplementary Figure S3), the severity of foliar symptoms in infected transgenic lines continued to increase until harvest at 12 MAP (Table 2).

**Table 2:** **CBSD incidence and severity in shoots and storage roots of transgenic p5001 TME 204 and controls plant lines at 6 and 12 months after planting (MAP) in a confined field trial conducted at Namulonge, Uganda**.

**Line/entry^a^**	**Shoot**		**Storage roots (12 MAP)**							
	**Av. severity score of CBSD symptoms (scale 1–5)**		**Number of plants harvested**	**Number of storage roots scored**	**Number. of storage roots showing CBSD symptoms^b^**		**Av. severity score of CBSD symptoms (scale 1–5)**	**Number of storage roots showing CBSD symptoms with score** >**2**		**Usable/Marketable storage roots (%)^c^**
	**6 MAP**	**12 MAP**			**Count**	**(%)**		**Count**	**%**	
5001-01	1.0	1.0	45	251	7	2.8	2.6	6.0	2.4	97.6
5001-08	1.0	1.0	47	327	6	1.8	2.0	2.0	0.6	99.4
5001-09	1.0	1.0	47	226	78	34.5	2.7	17.0	7.6	92.4
5001-10	1.0	1.0	49	373	22	5.9	2.1	13.0	3.5	96.5
5001-16	2.8	3.2	45	264	230	87.1	3.8	220.0	83.3	16.7
5001-18	1.0	1.0	43	265	21	7.9	2.2	9.0	3.4	96.6
5001-20	3.0	3.4	43	217	171	78.8	3.7	164.0	76.3	23.7
5001-22	2.5	2.5	46	279	8	2.9	2.2	2.0	0.7	99.3
5001-25	1.0	1.0	35	185	1	0.5	2.0	0.0	0.0	100
5001-26	1.0	1.0	47	215	3	1.4	3.5	1.0	0.5	99.5
5001-27	2.9	2.2	41	214	116	54.2	3.4	116.0	54.0	46.0
5001-30	1.0	1.0	44	251	1	0.4	2.0	0.0	0.0	100
5001-34	1.0	1.0	49	293	16	5.5	2.2	2.0	0.7	99.3
5001-35	1.0	1.0	43	211	9	4.3	2.8	6.0	2.8	97.2
5001-40	3.4	3.6	50	260	239	91.9	4.2	232.0	88.9	11.1
5001-42	1.0	1.0	50	331	9	2.7	2.2	3.0	0.9	99.1
5001-45	2.8	3.1	47	199	107	53.8	4.3	103.0	51.5	48.5
5001-46	1.0	1.0	48	270	3	1.1	2.0	0.0	0.0	100
5001-47	1.0	1.0	46	259	10	3.9	2.0	1.0	0.4	99.6
5001-48	3.0	3.5	47	219	143	65.3	3.8	142.0	64.8	35.2
5001-50	1.0	1.0	49	243	4	1.6	2.2	1.0	0.4	99.6
5001-53	3.0	3.2	44	255	93	36.5	3.7	71.0	28.5	71.5
5001-69	1.0	1.0	48	276	19	6.9	2.5	2.0	0.8	99.2
5001-76	3.0	3.0	43	235	34	14.5	3.9	26.0	11.5	88.5
5001-77	1.0	1.0	41	228	10	4.4	2.2	4.0	1.8	98.2
TME 204-WT1	2.6	3.2	49	265	264	99.6	4.2	200.0	79.1	20.9
TME 204-WT3	2.8	3.4	50	277	263	94.9	4.5	209.0	75.5	24.5
TME 204-WT3	2.9	3.4	44	219	219	100	4.4	222.0	100.0	0.0
NASE 3	3.4	4.3	23	46	41	89.1	4.5	39.0	84.8	15.2
NASE 14	4.1	4.4	29	89	78	87.6	4.0	72.0	80.0	20.0

MAP: months after planting

a *TME 204-WT1 and -WT2 represent entries of wild type TME 204 derived from in vitro micro-propagated plants. Entries TME 204-WT3, NASE 3, and NASE 14 wild-type plants were derived from disease-free stake cuttings obtained from farmers' fields*.

b *All storage roots were sliced transversely into five roughly equivalent sized segments and each slice assessed for presence and severity of CBSD symptoms on a scale of 1–5 (Ogwok et al., 2012)*.

c *Usable/marketable storage roots were assessed as those showing disease severity score of 1–2 on their slices*.

At 6.5 and 12 MAP, storage roots were harvested and assessed for CBSD-induced necrosis. At 6.5 MAP, three replicates were harvested and CBSD root damage assessed by slicing each storage root transversely five times along its length and scoring each slice for presence and severity of necrosis on a scale of 1–5 (Ogwok et al., 2012). Storage roots from all plants in all three entries of wild-type TME 204 displayed CBSD at 6.5 MAP, while local control cultivars NASE 3 and NASE 14 had incidences of CBSD at 75 and 100%, respectively (Supplementary Figure S3). In contrast, 16 of the 25 transgenic lines were found to have significantly lower averages of CBSD incidence with less than 20% of their plants carrying one or more storage roots showing CBSD symptoms (Supplementary Figure S3). At 12 MAP, the remaining five replicate plots were harvested and storage roots sliced and evaluated for presence and severity of CBSD. Eleven transgenic lines continued to have less than 20% of their plants (498 total plants examined) showing CBSD in one or more of their storage roots (Figure 1B). These transgenic lines showed average severity scores of between 2.0 and 2.7 (Table 2). In contrast, CBSD incidence in the wild-type TME 204 and local controls ranged between 80-100%, with average severity scores of 4.0-4.5 (Table 2, Figure 1B).

**Figure 2:**
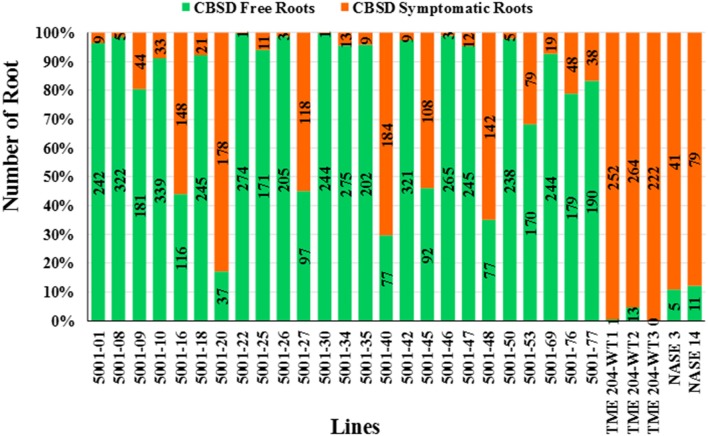
**Impact of CBSD on storage roots of cassava harvested from transgenic and non-transgenic plants 12 months after planting in a confined field trial at Namulonge, Uganda**. Twenty-five RNAi plant lines of cultivar TME 204 transgenic for the inverted repeat construct p5001 were assessed in addition to three wild-type entries of TME 204 (WT1, WT2, and WT3) plus non-transgenic local cultivars NASE 3 and NASE 14. At harvest, all storage roots were uprooted and sliced transversely five times along their length. A root was assessed positive for presence of CBSD if symptoms were visible on one or more of the slices. Total number of storage roots examined for each transgenic line is shown within the bar. Storage roots free of CBSD symptoms are indicated in green with those positive for presence of CBSD represented in red.

**Figure 3:**
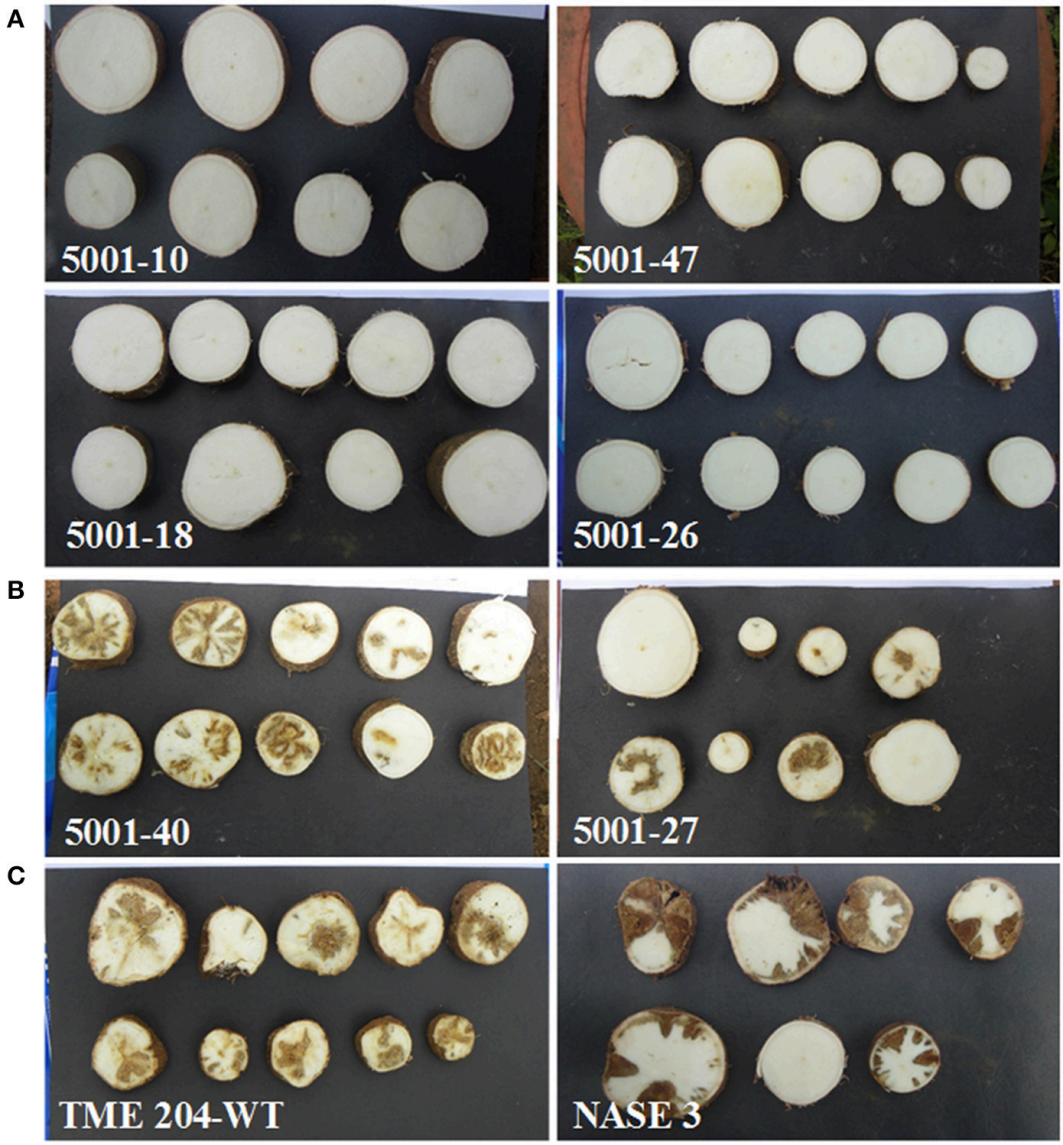
**CBSD symptoms within storage roots of cassava harvested from transgenic and non-transgenic plants 12 months after planting in a confined field trial at Namulonge, Uganda**. **(A)** Representative storage root slices from high CP-derived siRNA-accumulating transgenic lines 5001-10, 47, 18, and 26 showing no CBSD symptoms **(B)** storage root slices from representative low CP-derived siRNA accumulating lines 5001-40 and 5001-27 showing CBSD symptoms, **(C)** root slices of CBSD symptomatic wild-type TME 204 (left) and non-transgenic local cultivar NASE 3 (right) showing severe symptoms of CBSD.

A total of 7242 storage roots were evaluated at 12 MAP (6346 roots from the transgenic lines and 896 roots from wild-type controls). The number of roots per plant line showing CBSD symptoms is presented in Figure 3. In 16 of the 25 transgenic lines, over 90% of the storage roots remained free from CBSD symptoms. For 12 of these lines, less than 5% of the storage roots displayed CBSD. In contrast, the three entries of wild-type TME 204 had 95-100% of their storage roots symptomatic for CBSD, with only 14 roots free of CBSD out of a total of 761 roots assessed (Figure 2, Table 2). The number of roots showing CBSD symptoms in the local cultivars NASE 3 and NASE 14 was also high at 87–89% (Figure 3).

Storage roots carrying very mild CBSD symptoms still have value and can be consumed. In order to determine the impact of CBSD on marketable/usable root yields, CBSD symptom severity data was re-analyzed, defining usable storage roots as those with scores of 1–2 on the severity scale of 1–5, similar to breeders' practices employed for line selection (Kawuki et al., 2016). Based on this assessment, the wild-type TME 204 and local controls had between 0% and 25% usable storage roots at 12 MAP while 11 of the 25 transgenic TME 204 lines had 98–100% of their roots defined as usable (Table 2).

Populations of adult whitefly counted on the five uppermost leaves steadily increased from planting to a maximum average of 310 per plant at four MAP and declining to less than 10 whiteflies per plant at 11 MAP (Supplementary Figure S2). There was no vector preference observed within transgenic lines, or between the transgenic and non-transgenic TME 204 control plants and local varieties (Supplementary Figure S2).

### Correlation of resistance to CBSD with expression of transgene-derived siRNA

**Figure 4:**
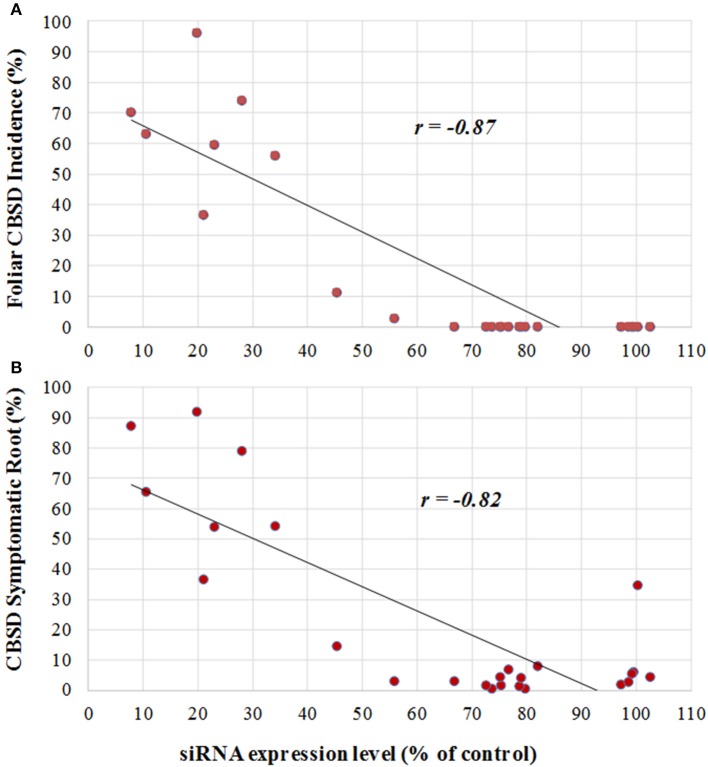
**Correlation between levels of transgene-derived siRNA expression and visual incidence of CBSD symptoms in TME 204 plant lines 12 months after planting in a confined field trial at Namulonge, Uganda**. Twenty-five plant lines for the inverted repeat construct p5001 were assessed for levels of transgene-derived siRNA accumulation by Northern blot analysis from *in vitro*-grown plants before establishment in the field. Signal intensities of the small RNA blots were analyzed using ImageJ software and values were normalized against the known high siRNA-expressing cassava plant line 718-01 (Ogwok et al., 2012). Northern blot numerical expression values of each line were plotted against CBSD incidence. **(A)** Relationship between UCBSV-derived siRNA expression with foliar CBSD incidence at 12 MAP, **(B)** relationship between UCBSV-derived siRNA expression with percentage of CBSD symptomatic storage root at 12 MAP.

Levels of transgene-derived (CBSV-CP and UCBSV-CP) siRNAs accumulating within tissues of the 25 p5001 lines cultivated in the CFT at Namulonge were determined before plants were established in the field (Supplementary Figure S1). Incidence of CBSD symptoms directly correlated with levels of accumulated CP-derived siRNAs in the transgenic lines. Plant lines that accumulated medium to high levels of transgene CP-derived siRNAs displayed very high resistance against CBSD. These lines displayed no foliar symptoms and had very low incidence of symptomatic storage roots (Figures 1–3). Transgenic TME 204 lines classified as low siRNA expressers (5001-16, 5001-20, 5001-27, 5001-40, 5001-45, and 5001-48) showed lower resistance to CBSD under field conditions at Namulonge (Figures 1–3). This was reflected by CBSD incidence in shoots and storage roots and in the proportion of marketable storage roots. A strong Pearson correlation (*r* = −0.87) was observed between levels of transgene-derived siRNA expression level and foliar CBSD incidence (Figure 4A), and likewise between transgene derived siRNA expression and percentage of CBSD symptomatic storage roots (*r* = −0.84, Figure 4B). The most resistant lines showed the highest levels of CP-derived siRNA expression.

### p5001-imparted resistance across the vegetative cropping cycle

Durability of resistance to CBSD imparted by p5001 was assessed across the vegetative cropping cycle by establishing a CFT at Namulonge with stake cuttings obtained from the initial CFT described above. Stem cuttings were generated from plants harvested at 6.5 MAP. Eleven transgenic lines showing no visual foliar CBSD symptoms and minimum root CBSD incidences (Table 1, Supplementary Figure S3) were selected, planted and grown for a period of 8 months. In addition, controls were established from stem cuttings obtained from CBSD- and CMD-free plants of TME 204 obtained from a farmer's field.

Whereas very high whitefly populations were observed within the initial CFT established from tissue culture-derived plants, insect counts remained relatively low in the durability trial with peak average populations of 19 per plant by two MAP (data not shown). However, more than 61% of TME 204 plants established from disease-free stem cuttings developed CBSD on their foliar tissues by 8 MAP (shown as WT2 in Supplementary Table S1), indicating that new infections were being vectored into this CFT. All p5001 transgenic TME 204 plants remained free of visible CBSD foliar symptoms over the eight-month growing period (Supplementary Table S1).

**Figure 5:**
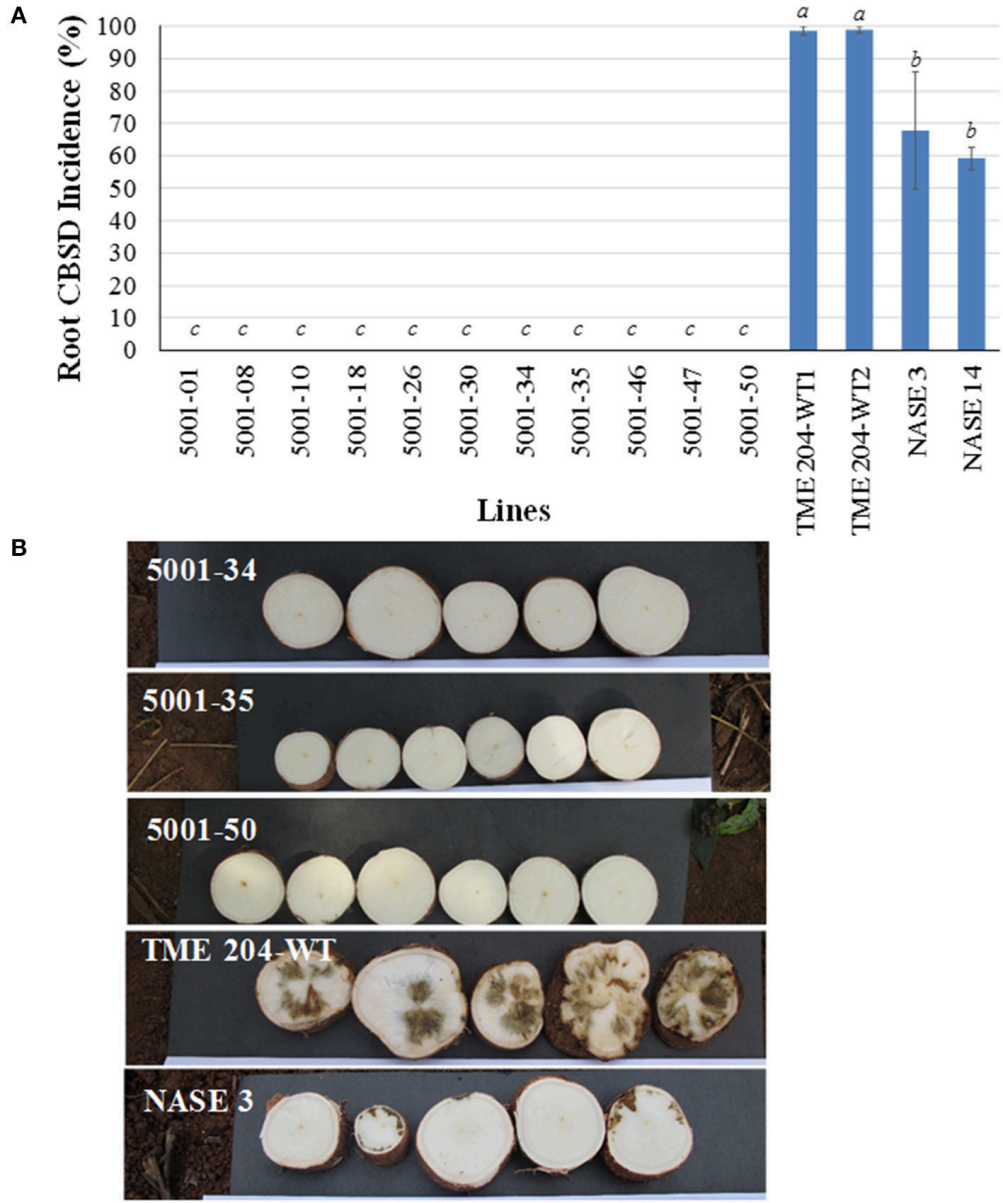
**Incidence of CBSD symptoms in storage roots of transgenic TME 204 p5001 lines, non-transgenic wild-type TME 204 and local controls in a stake-derived field trial at Namulonge, Uganda**. Stem cuttings obtained from the first trial at Namulonge (Figures 1–3) were used to establish a second trial to determine durability of p5001-imparted RNAi resistance to CBSD. Plants of TME 204-WT2 were established from disease-free cuttings obtained from a farmer's field. **(A)** Incidence of CBSD symptoms in storage roots at 8 months after planting. Data per line represents average number of plants showing CBSD symptoms in their storage roots expressed as percentage. Means followed by same letter do not significantly differ (*P* ≤ 0.05, Duncan's multiple range test). **(B)** CBSD symptoms within storage roots of representative transgenic lines 5001-34, 5001-35, and 5001-50; symptomatic wild-type TME 204; and non-transgenic local control NASE 3.

At 8 MAP, storage roots were harvested and assessed by transverse slicing as described above to determine levels of CBSD-induced damage. None of the 11 transgenic p5001 lines had CBSD symptomatic roots (Figures 5A,B), and all harvested storage roots were therefore classified as usable. Both TME 204 non-transgenic entries developed storage root CBSD incidences of 99% (Figure 5) and average severity scores of 3.9–4.1 (Supplementary Table S1). The local controls NASE 3 and NASE 14 were also severely impacted by CBSD, displaying incidences at 68 and 59%, respectively (Figure 5) with average severity scores of 3.6 and 3.4 (Supplementary Table S1).

### RT-PCR detection of CBSD causal viruses in the CFTs

Presence of CBSV and UCBSV within the CFTs was determined using species-specific primers (Mbanzibwa et al., 2011). Pools of five leaf samples obtained from asymptomatic plants were analyzed at 7 MAP by RT-PCR. All asymptomatic transgenic lines were seen to be free of detectable viruses in both the initial trial and the stake- derived trial (Table 3). Wild-type TME 204 control samples, in contrast, were found to carry both CBSV and UCBSV, present either as single or dual infections in up to 87.5% of the samples analyzed (Table 3).

**Table 3:** **Reverse transcriptase-polymerase chain reaction (RT-PCR) diagnostics for simultaneous detection of ***Cassava brown streak virus*** (CBSV) and ***Ugandan Cassava brown streak virus*** (UCBSV) at 7 months after planting in confined trials conducted at Namulonge, Uganda**.

**Line/entry^a^**	**Trial established from tissue culture-derived plantlets**					**Durability trial established from stake-derived cuttings**			
	**Number of samples analyzed^b^**	**Positive samples (%)**	**Only CBSV (%)**	**Only UCBSV (%)**	**CBSV & UCBSV (%)**	**Positive samples (%)**	**Only CBSV (%)**	**Only UCBSV (%)**	**CBSV & UCBSV (%)**
5001-01	8	0	0	0	0	0	0	0	0
5001-08	8	0	0	0	0	0	0	0	0
5001-09	8	0	0	0	0	n/a	n/a	n/a	n/a
5001-10	8	0	0	0	0	0	0	0	0
5001-18	8	0	0	0	0	n/a	n/a	n/a	n/a
5001-25	8	0	0	0	0	n/a	n/a	n/a	n/a
5001-26	8	0	0	0	0	0	0	0	0
5001-30	8	0	0	0	0	0	0	0	0
5001-34	8	0	0	0	0	0	0	0	0
5001-35	8	0	0	0	0	0	0	0	0
5001-42	8	0	0	0	0	n/a	n/a	n/a	n/a
5001-46	8	0	0	0	0	0	0	0	0
5001-47	8	0	0	0	0	0	0	0	0
5001-50	8	0	0	0	0	0	0	0	0
5001-69	8	0	0	0	0	0	0	0	0
5001-77	8	0	0	0	0	n/a	n/a	n/a	n/a
TME 204-WT1	8	87.5	37.5	12.5	37.5	87.5	12.5	12.5	50
TME 204-WT2	8	87.5	12.5	12.5	62.5	87.5	25	37.5	37.5

a *-WT1 and -WT2 represent entries of wild-type TME 204 derived from tissue culture micro-propagated plants. TME 204-WT2 in durability trial is established from locally sourced stake cuttings. N/A, denotes samples not assayed*.

b *Leaves from five randomly selected plants were sampled per plot and pooled for RNA extraction and virus diagnostics*.

### Performance of transgenic p5001 lines at Mtwapa, Kenya

Performance of TME 204 plants expressing the p5001 RNAi construct was assessed at Mtwapa, coastal Kenya, to determine their resistance to CBSD under different agro-ecological conditions from that present at Namulonge. The Mtwapa CFT was established with a subset of 19 of the 25 lines tested at Namulonge, plus two entries of non-transgenic TME 204. The 19 transgenic lines were chosen to include 14 that expressed medium to high, and five that expressed low levels of transgenic CP-derived siRNAs (Table 1, Supplementary Figure S1). By 12 MAP, 10 of the 19 transgenic lines had developed visible foliar CBSD symptoms with incidences ranging between 3–8% and average severity scores of 2.0–3.0 (Table 4). Foliar CBSD symptoms of the two wild-type entries were seen at 100% with average severity scores at or close to 3.0 (Table 4).

**Table 4:** **CBSD incidence and severity in shoots and storage roots of transgenic p5001 TME 204 and control plant lines at 6 and 12 months after planting (MAP) in a confined field trial conducted at Mtwapa, Kenya**.

**Line/entry^a^**	**Shoot**				**Storage roots (12 MAP)**							
	**CBSD incidence (%)**		**Av. severity score of CBSD symptoms (scale 1–5)**		**Number of plants harvest-ed**	**Number of storage roots scored**	**Number of storage roots showing CBSD symptoms^b^**		**Av. severity of CBSD symptoms (scale 1–5)**	**Number of storage roots showing CBSD symptoms with score** >**2**		**Usable/Marketable storage roots (%)^c^**
	**6 MAP**	**12 MAP**	**6 MAP**	**12 MAP**			**Count**	**(%)**		**Count**	**%**	
5001-01	0.0	3.0	1.0	2.0	40	216	26	12.0	2.3	4.0	1.9	98.1
5001-08	0.0	0.0	1.0	1.0	40	251	32	12.7	2.0	1.0	0.4	99.6
5001-10	0.0	0.0	1.0	1.0	40	255	22	8.6	2.1	5.0	2.0	98
5001-16	0.0	3.0	1.0	2.0	40	259	28	10.8	2.2	10.0	3.9	96.1
5001-18	3.0	3.0	2.0	2.0	40	261	6	2.3	2.5	1.0	0.4	99.6
5001-20	5.0	5.0	2.0	2.0	40	223	20	9.0	2.6	10.0	4.5	95.5
5001-26	0.0	0.0	1.0	1.0	40	253	18	7.1	2.0	2.0	0.8	99.2
5001-30	0.0	0.0	1.0	1.0	40	241	20	8.3	2.0	0.0	0.0	100
5001-34	0.0	0.0	1.0	1.0	40	237	10	4.2	2.1	1.0	0.4	99.6
5001-35	0.0	0.0	1.0	1.0	40	239	11	4.6	2.0	0.0	0.0	100
5001-40	8.0	8.0	2.0	2.3	40	237	31	13.1	2.9	18.0	7.6	92.4
5001-42	3.0	5.0	2.0	2.0	40	234	13	5.6	2.1	4.0	1.7	98.3
5001-46	5.0	8.0	2.0	2.0	40	232	21	9.1	2.1	3.0	1.3	98.7
5001-47	0.0	5.0	2.0	2.0	40	229	8	3.5	2.1	2.0	0.9	99.1
5001-50	3.0	5.0	2.0	2.0	40	270	12	4.4	2.3	5.0	1.9	98.1
5001-53	0.0	0.0	1.0	1.0	40	240	13	5.4	2.1	1.0	0.4	99.6
5001-69	0.0	0.0	1.0	1.0	40	251	12	4.8	2.1	2.0	0.8	99.2
5001-76	0.0	3.0	1.0	3.0	40	248	15	6.0	2.1	1.0	0.4	99.6
5001-77	0.0	0.0	1.0	1.0	40	246	6	2.4	2.1	1.0	0.4	99.6
TME 204-WT1	60.0	100.0	2.0	2.9	40	253	242	95.7	4.0	226.0	89.3	10.7
TME 204-WT2	40.0	100.0	2.0	3.0	40	280	243	86.8	3.6	214.0	76.4	23.6

*MAP: months after planting*.

a *TME 204-WT1, and -WT2 represent entries of wild-type TME 204 derived from tissue culture micro-propagated plants*.

b *All storage roots were sliced transversely into five roughly equivalent sized segments and each slice assessed for presence and severity of CBSD symptoms on a scale of 1–5 (Ogwok et al., 2012)*.

c *Usable/marketable storage roots were assessed as those showing disease severity score of 1–2 on their slices*.

**Figure 6:**
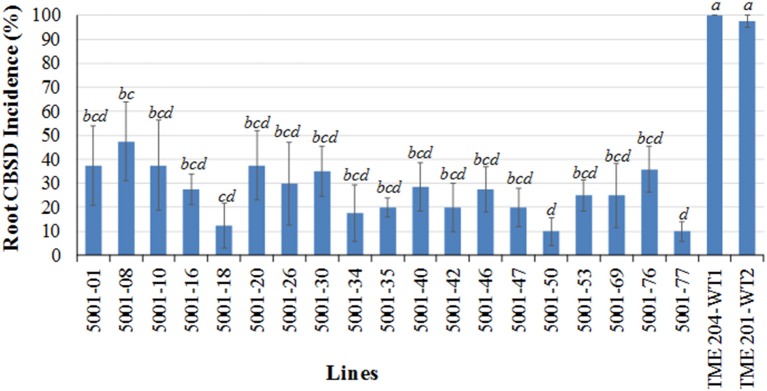
**Incidence of CBSD symptoms in storage roots of transgenic TME 204 plant lines and non-transgenic TME 204 controls at 12 months after planting within a confined field trial at Mtwapa, Kenya**. The 19 transgenic TME 204 lines studied were generated using the p5001 construct. TME 204-WT1 and -WT2 are two entries of wild-type TME 204 derived from *in vitro* micro-propagated plants. Data were collected from four replicated plots of 10 plants each. Storage roots were sliced five times along their length and visually assessed for presence of CBSD symptoms. Data is presented as the average percentage of plants showing CBSD symptoms across the four plots. Approximately five storage roots were assessed per plant. Presence of CBSD within one or more storage roots resulted in a positive score of that plant. Means followed by the same letter are not significantly different (*P* ≤ 0.05, Duncan's multiple range test).

Storage roots from a total of 40 plants per line (4 replicates of 10 plants each) were harvested at 12 MAP and evaluated for presence and severity of CBSD symptoms. The frequency of plants showing CBSD symptoms in their storage roots ranged between 10.0 and 47.5% in the transgenic p5001 lines with seven of these lines showing incidences of 10–20% (Figure 6). The two wild-type entries of TME 204 had CBSD incidences of 97.5–100% (Figure 6).

**Figure 7:**
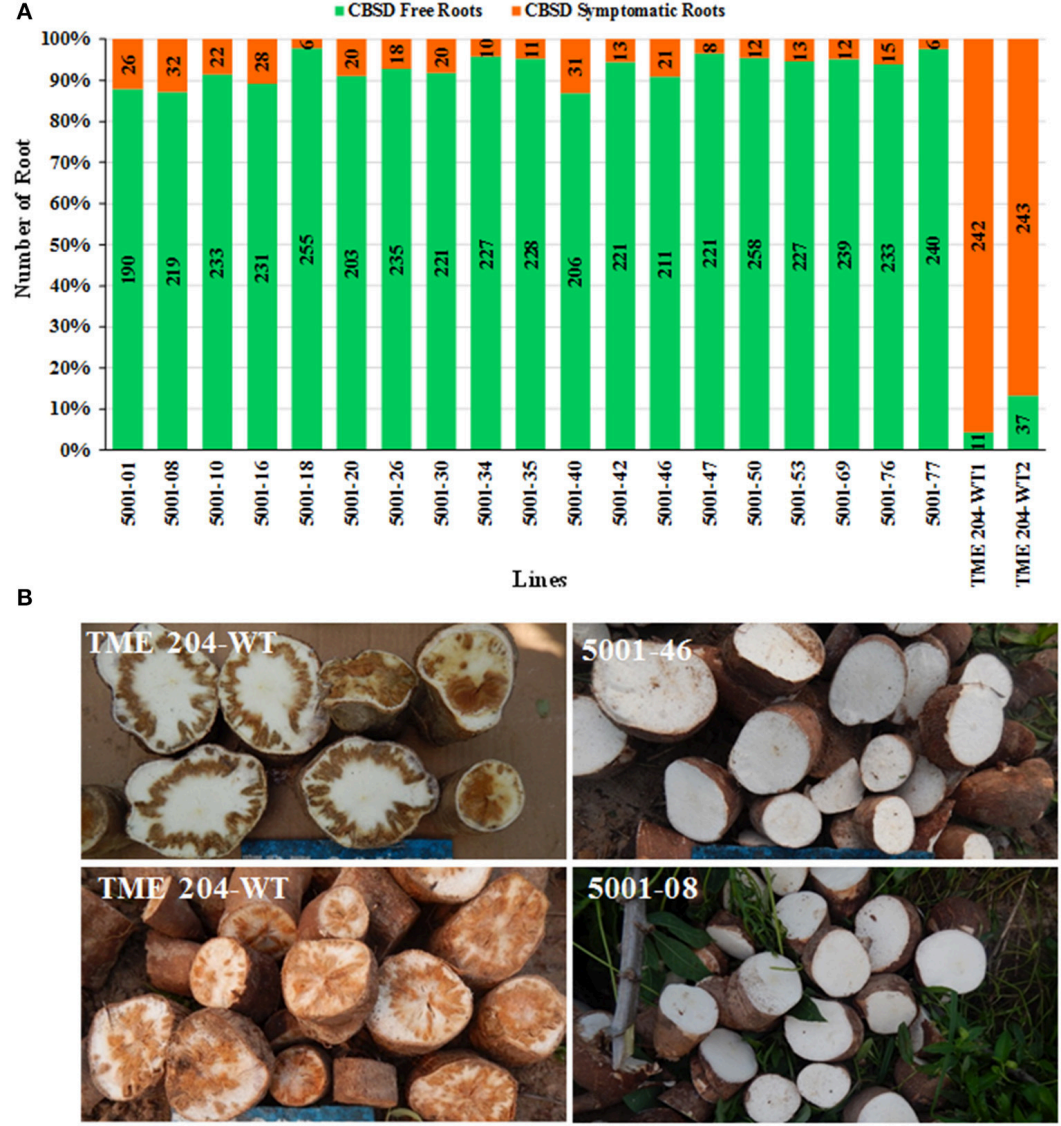
**Impact of CBSD on storage roots of cassava harvested from transgenic and non-transgenic plants 12 months after planting in a confined field trial at Mtwapa, Kenya. (A)** Nineteen RNAi plant lines of cultivar TME 204 transgenic for the inverted repeat construct p5001 were assessed in addition to two wild-type entries of TME 204 (-WT1, -WT2). At harvest, all storage roots were uprooted and sliced five times along their length. A root was assessed positive for presence of CBSD if symptoms were visible on one or more of the slices. Total number of storage roots examined for each transgenic line is shown within the bar. Storage roots free of CBSD symptoms are indicated in green with those positive for presence of CBSD represented in red. **(B)** Representative storage root slices from plants of non-transgenic TME 204 (upper and lower left) and two RNAi transgenic plant lines (upper and lower right).

The total number of roots per line showing CBSD symptom was also determined across the total of 5155 storage roots evaluated. Storage roots showing any CBSD symptoms (score greater than 1 in 1–5 scale) ranged between 2.5–13.5% for the 19 transgenic lines, of which 15 had less than 10% of their storage roots symptomatic for CBSD (Figures 7A,B). This compared to 87–96% symptomatic storage roots for the two entries of wild-type TME 204. Evaluation of the marketable/usable roots was assessed by counting all roots with CBSD severity scores of 2 or less. Non-transgenic wild-type TME 204 had only 10–24% of their storage roots assessed to be usable at 12 MAP. In contrast, 15 of the 19 transgenic lines had usable yield losses of 2% or less when assessed in this manner (Table 4).

### Sequence diversity of CBSV and UCBSV at the CFT sites

**Figure 8:**
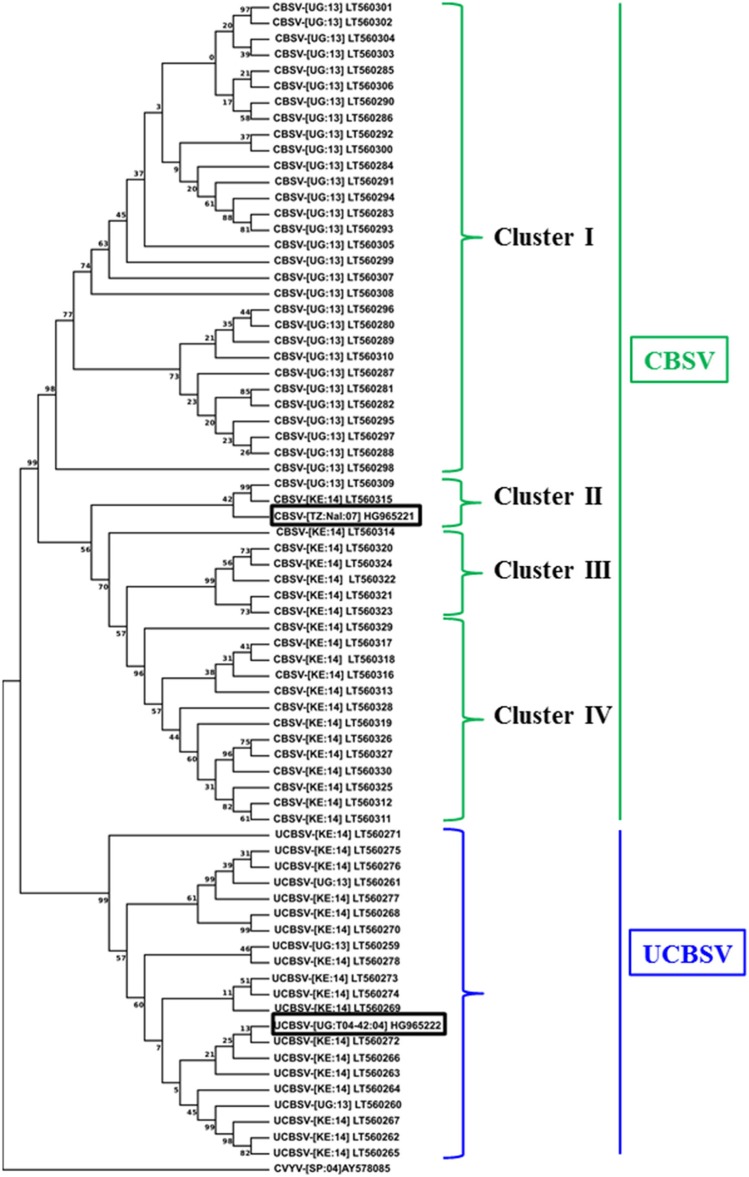
**Phylogenetic analysis of CBSV and UCBSV based on alignment of nucleotide sequences derived from the 3′-end of the virus genome, including the full-length CP regions**. Virus samples were collected from within the field trial site and nearby plots in Mtwapa, Kenya and within the field trials at Namulonge, Uganda in 2013 and 2014. The numbers at nodes are percentage bootstrap confidence scores (1000 replicates). The tree was arbitrarily rooted on the cognate sequence of *Cucumber vein yellowing virus* (AY578085). The 3′ genomic region of the two reference isolates (rectangular boxes) from which coat protein nucleotide sequences were obtained for making the p5001 inverted repeats CBSV isolate CBSV-[TZ:Nal:07] GenBank HG965221: 7331-8937 and UCBSV isolate UCBSV-[UG-T04-42:04] GenBank HG965222: 7315-8984 were included for comparison. Nucleotide sequences of clones identified in this study are deposited in public database with accession numbers (GenBank LT560259-LT560330).

The genetic diversity of CBSV and UCBSV viruses infecting cassava plants within and surrounding the CFT locations at Namulonge and Mtwapa was investigated. Over 100 samples were obtained as dried pressed leaves, processed, cloned and sequenced. The sequenced fragments identified 34 and 38 non-redundant isolates from Namulonge and Mtwapa, respectively. A BLAST search (NCBI) showed that at Namulonge, 31 out of the 34 isolates were CBSV, with the remaining three identified as UCBSV. Unlike Namulonge, isolates from Mtwapa were more evenly balanced between CBSV and UCBSV, with 20 samples corresponding to CBSV and 17 to UCBSV. Sequence alignments and analysis showed that the CBSV sequences were 86–99% identical at nucleotide level. A phylogenetic analysis sub-divided the CBSV into four clusters with sequences in each cluster having distinct geographical origins (Figure 8). Cluster I contained 30 sequences with nucleotide identity ranging between 97 and 99%. This cluster was the largest of the four, and contained isolates from the Ugandan site alone. These isolates were 90–91% identical to the CBSV sequence (CBSV[TZ:Nal3-1:07, GenBank HG965221) used to generate the inverted repeat expression cassettes present in p5001. Cluster III and IV consisted of CBSV isolates from Mtwapa, Kenya. Sequences from cluster III were 99% identical to each other, and 92% identical to the p5001 reference sequence HG965221 at the nucleotide level. Sequences in cluster IV were 96–99 and 90–93% similar to each other and the reference sequence, respectively. Cluster II contained only two isolates collected from Namulonge and Mtwapa and were 93% identical to the CBSV HG965221 reference sequence. All 20 UCBSV isolates (three from Namulonge and 17 from Mtwapa) clustered together with sequence identity that ranged between 92–98%, and had 92–98% similar identity to the reference sequence (UCBSV[UG:T04-42:04, GenBank HG965222) used to generate the inverted repeat in the p5001 inverted repeat construct (Figure 8).

## Discussion

Cassava cultivar TME 204 was genetically modified to express siRNAs derived from the inverted repeat construct p5001 consisting of fused CP sequences from CBSV and UCBSV (Chauhan et al., 2015; Beyene et al., 2016a). TME 204 is a Ugandan farmer-preferred cultivar possessing good root characteristics, early maturation time and robust resistance to CMD. It is, however, highly susceptible to CBSD. These qualities make TME 204 an ideal target for improvement within the VIRCA project, which aims to bring CMD- and CBSD-resistant planting material to smallholder farmers in East Africa (Taylor et al., 2012). We previously reported field resistance to CBSD generated from an inverted repeat construct carrying the CP of UCBSV (p718) (Ogwok et al., 2012; Odipio et al., 2014). While high levels of resistance to the homologous virus species was achieved in p718 transgenic plants, resistance to CBSV was restricted to two lines that accumulated CP-derived siRNAs at very high levels (Ogwok et al., 2012). Susceptibility of these lines to CBSV was confirmed by Beyene et al. (2016a), when p718 transgenic plants were bud graft inoculated with CBSV. Recognizing the need for integration of more robust resistance against both CBSD causal viruses, plants of TME 204 were generated with the p5001 construct (Chauhan et al., 2015; Beyene et al., 2016a). Proof of efficacy under greenhouse conditions against inoculation with CBSV and UCBSV led to the establishment of a field trial program in East Africa where the plants were exposed to the pathogens transmitted by whitefly vector.

Field performance of p5001 transgenic plants against CBSD exceeded expectations. Under field conditions at Namulonge, Uganda, in which 95–100% of non-modified TME 204 plants developed symptoms, 16 of the 25 lines developed no foliar symptoms and showed significantly reduced (<20%) CBSD symptom incidences in storage roots (Figures 1–3) over the 12-month growing period. Similar results were obtained in Mtwapa where 7 of the 19 lines tested displayed 10–20% CBSD incidences in their storage roots. Efficacy of RNAi-imparted resistance was most dramatically seen when comparing the impact of CBSD on usable storage root yields, defined as roots carrying no more than a disease severity score of 2 (scale 1–5). In this case more than 95% of storage roots produced by the non-transgenic TME 204 were lost to disease (score of 3 or more). Under the same conditions in both central Uganda and coastal Kenya, the best performing transgenic lines lost no more than 2% of their roots to damage from CBSD (Figures 2, 7, Tables 2, 4).

In all cases the best performing plant lines were those that accumulated higher levels of transgene CP-derived siRNAs. Greenhouse studies indicated a positive correlation between levels of p5001 CP-derived siRNAs and resistance to challenge with CBSV and UCBSV (Beyene et al., 2016a). The 25 p5001 transgenic lines selected for testing under CFT conditions confirmed that this phenomenon translates to the field, and that levels of siRNA determined from plantlets as early as 4 weeks after regeneration in tissue culture can be used as a signature for subsequent vector-transmitted field-level performance against the virus pathogens (Figures 1–6). This has important implications for streamlining product development, allowing effective selection for CBSD-resistant plant lines as early as the newly regenerated *in vitro* plantlet. Only high CP-derived siRNA accumulating lines need then be taken through the pipeline to field-testing stages. Likewise, mid-season assessments of storage roots (6.5 MAP) tracked consistently well with end of season p5001 TME 204 performance (strong age to age correlation) indicating the possibility of early phenotype screening and identification of adapted lines in high CBSD disease locations.

The overall incidence and severity of CBSD on transgenic lines seen at Mtwapa was lower than that recorded at Namulonge (Figures 1–7, Tables 2, 4). Impact of CBSD remained high, with greater than 90% of non-transgenic storage roots impacted by disease. Across the 10 transgenic lines, all but two recorded presence of CBSD to be less than 10% of their roots (Figure 7). The resistance conferred by p5001 and ranking of events accumulating lower levels of CP-derived siRNAs at this location may suggest differences in virulence of the CBSD causal pathogens and/or a combination of the environment and CBSD pressure. Year-to-year variation in CBSD incidence and severity has been reported at the same location (Kawuki et al., 2016). Indeed, data generated from the diversity study reported here indicates presence of a cluster of CBSV isolates at Namulonge (Cluster I) (Figure 8) that was not found at the Mtwapa location (Figure 8).

Successful deployment of CBSD-resistant planting materials depends not just on levels of resistance to the pathogen, but also durability of the trait over cropping cycles, and the geographical range in which effective resistance can be maintained. Highly specific pathogen-derived resistance restricted to a narrow agro-ecological area, as seen for *Papaya ringspot virus* in Hawaii (Tennant et al., 1994), would prevent deployment of RNAi technology in cassava due to the dynamic nature of the disease complex and its insect vector across East and Central Africa (Legg et al., 2014; Ndunguru et al., 2015; Patil et al., 2015). Effective siRNA-derived resistance to CBSD across the vegetative cropping cycle reported here (Figure 5) demonstrates durability of the trait under practices similar to those used by farmers. Indeed, data presented showed that resistance in the stake-derived generation was equal or superior to that of tissue culture-derived plants at the same location (Figures 1–3). Proof of resistance to CBSD in diverse locations in central Uganda and coastal Kenya indicates that plants expressing siRNAs from the p5001 construct would have value across a significant part of East Africa. Efficacy of the p5001 transgenic lines in these diverse locations is possibly due to abundance of the transgene-derived siRNA produced in the resistant lines and the high CP sequence similarity (92.8–99.6% for UCBSV and 91.3–94.1% for CBSV) between the CP sequences of the transgene and the isolates present across these two CFT locations (Supplementary Tables S2, S3). It remains unclear if all regions within the CP sequences provide siRNAs that impart resistance to CBSD, or if this is restricted to specific regions.

Successful demonstration of RNAi for control of CBSD reported here would normally lead to the best performing lines being advanced for subsequent yield and regulatory CFTs. As reported previously (Beyene et al., 2016b), however, all 25 transgenic TME 204 plant lines were found to be susceptible to CMD under field conditions at Namulonge and Mtwapa. Extensive experimentation has since confirmed that loss of resistance to CMD was not as a result of p5001-derived siRNA expression, but occurred during the earliest steps in the induction of the embryogenic tissues employed for production of these and other transgenic cassava plants (Chauhan et al., 2015; Beyene et al., 2016b). Two parallel approaches are being pursued to circumvent this unforeseen phenomenon and capitalize on the proven efficacy of p5001 for imparting resistance to CBSD. Firstly, in order to generate stacked resistance to both CMD and CBSD in the resulting progeny, p5001 TME 204 transgenic plants are being crossed with non-transgenic CMD2-, CMD1- and CMD3-type cassava cultivars. Secondly, p5001 is being integrated into CMD1-and CMD3-type cultivars via new *Agrobacterium*-mediated transformations based on knowledge that these cultivars do not lose resistance to CMD when passed through somatic embryogenesis (Beyene et al., 2016b). Efficacy of RNAi to control CBSD has been demonstrated through a series of greenhouse (Beyene et al., 2016a) and field studies, the latter in diverse geographical regions of high disease pressure. Similar resistance to virus disease and subsequent regulatory approval for planting by farmers has been achieved in papaya (Fuchs and Gonsalves, 2007), squash (Gottula and Fuchs, 2009), plum (Hily et al., 2004; Scorza et al., 2013), and common bean (Aragao et al., 2013). Targeting introgression of this technology into cassava germplasm with inherent resistance to CMD therefore holds potential for delivering CBSD-resistant planting materials to farmers and breeders in East and Central Africa, and as a preemptive measure against likely progression of this destructive disease into West Africa.

## Author contributions

TA, AB, DM, NT, and MH conceived and planned and supervised the work. HW, GB, JA, JO, GO, RC, TM, MI, and HO performed the field and lab work and collected data. HW, GB, MI, and PR analyzed the data. HW, GB and NT wrote the manuscript. All authors contributed critically to the manuscript.

## Funding

Funding for this research was provided by the Bill and Melinda Gates Foundation (OPPGD1485), the United States Agency for International Development from the American people (USAID Cooperative Agreement No. AID-EDH-A-00-09-00010), and the Monsanto Fund.

### Conflict of interest statement

The authors declare that the research was conducted in the absence of any commercial or financial relationships that could be construed as a potential conflict of interest.
