# Supplementary material for: Field Level RNAi-Mediated Resistance to Cassava Brown Streak Disease across Multiple Cropping Cycles and Diverse East African Agro-Ecological Locations
Source: Front Plant Sci. 2017 Jan 12;7:2060. doi: 10.3389/fpls.2016.02060 (PMC5226948; doi:10.3389/fpls.2016.02060)
Supplement: Supplementary file 1 [file Table1.docx]

**Field level RNAi-mediated resistance to Cassava brown streak disease across multiple cropping cycles and diverse East African agro-ecological locations**

Henry Wagaba, Getu Beyene, Jude Aleu, John Odipio, Okao-Okuja Geoffrey, Raj Deepika Chauhan, Theresia Munga, Hannington Obiero, Mark Halsey, Muhammad Ilyas, Peter Raymond, Anton Bua, Nigel J. Taylor*, Douglas Miano and Titus Alicai

* Correspondence: Nigel Taylor: Ntaylor@danforthcenter.org

**Supplementary Table S1**. CBSD incidence and severity in shoots and storage roots of transgenic p5001 TME 204, wild type TME 204 and local controls at eight months after planting in stake-derived durability trial conducted at Namulonge, Uganda.

| **Line** | **Shoot CBSD** | | **Root CBSD** | |
| --- | --- | --- | --- | --- |
|  | **Incidence** (%) | **Ave. Severity** (1-5) | **Incidence** (%) | **Ave. Severity** (1-5) |
| 5001-01 | 0 | 1 | 0 | 1 |
| 5001-08 | 0 | 1 | 0 | 1 |
| 5001-10 | 0 | 1 | **0.02** | 1 |
| 5001-18 | 0 | 1 | 0 | 1 |
| 5001-26 | 0 | 1 | 0 | 1 |
| 5001-30 | 0 | 1 | 0 | 1 |
| 5001-34 | 0 | 1 | 0 | 1 |
| 5001-35 | 0 | 1 | 0 | 1 |
| 5001-46 | 0 | 1 | 0 | 1 |
| 5001-47 | 0 | 1 | 0 | 1 |
| 5001-50 | 0 | 1 | 0 | 1 |
| TME 204-WT1 | 100 | 3 | 86.1 | 4.1 |
| TME 204-WT2 | 61.2 | 3.3 | 93.3 | 3.9 |
| NASE 3 | 48 | 3.3 | 48.9 | 3.6 |
| NASE 14 | 69 | 4 | 25.8 | 3.4 |
