# Supplementary figures and images for: Field Level RNAi-Mediated Resistance to Cassava Brown Streak Disease across Multiple Cropping Cycles and Diverse East African Agro-Ecological Locations
Source: Front Plant Sci. 2017 Jan 12;7:2060. doi: 10.3389/fpls.2016.02060 (PMC5226948; doi:10.3389/fpls.2016.02060)

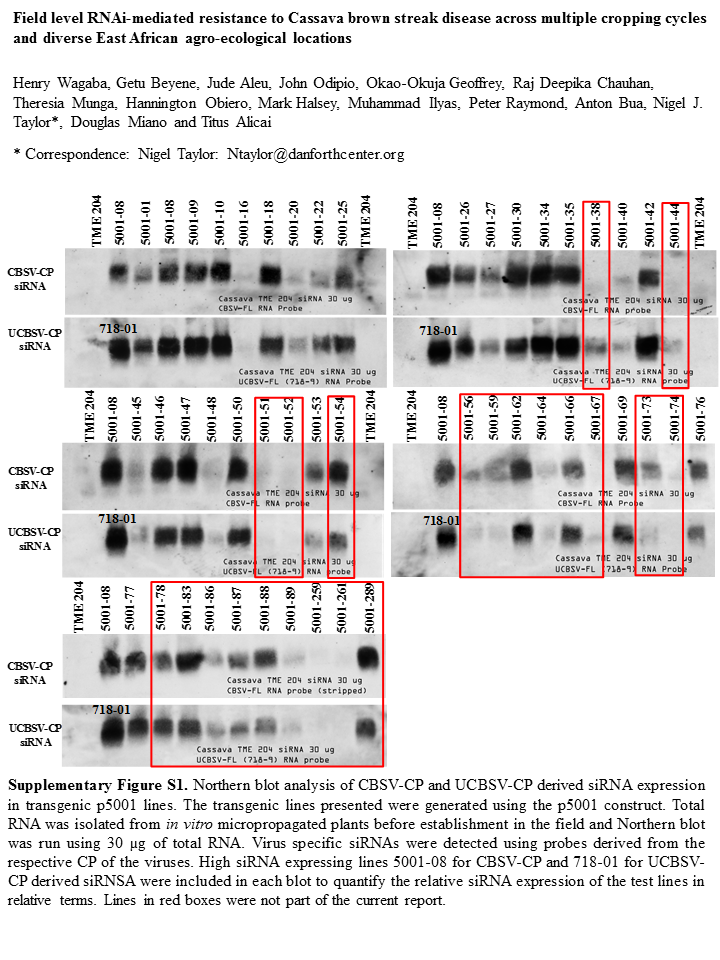

Supplement: Supplementary file 4 [file Image1.tif]

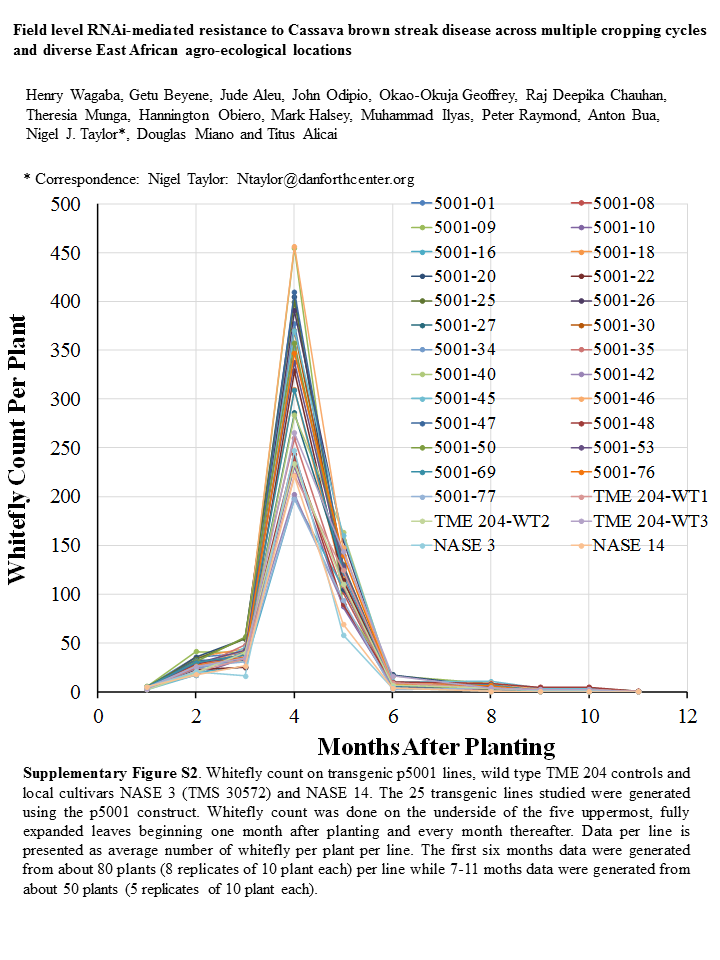

Supplement: Supplementary file 5 [file Image2.tif]

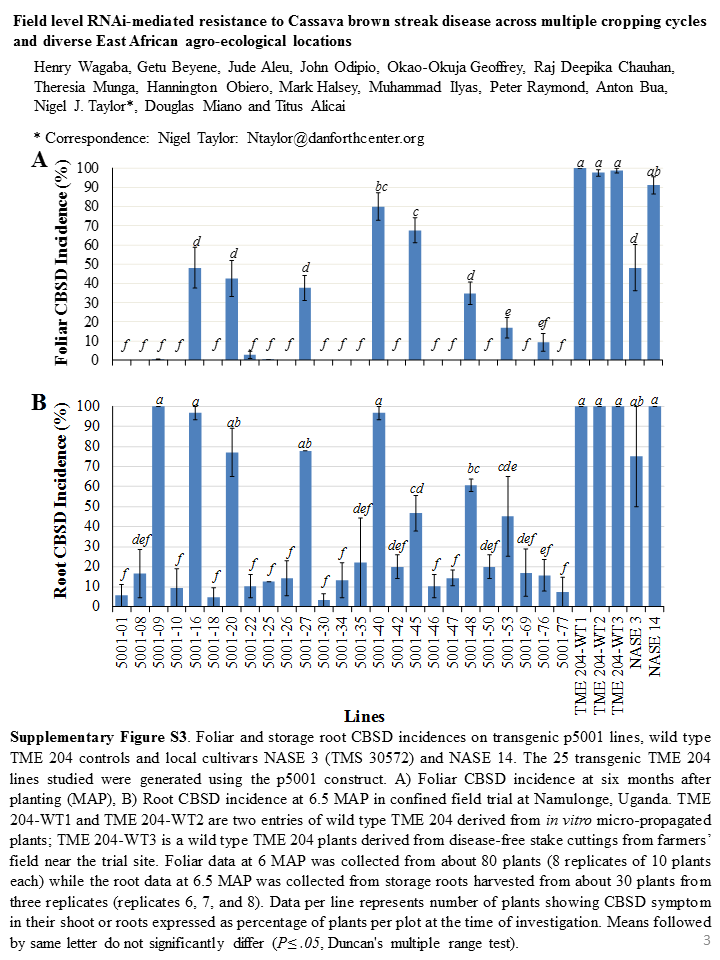

Supplement: Supplementary file 6 [file Image3.tif]
